# Trace metal accumulation with age in bats: a case study on *Pipistrellus kuhlii lepidus* from a relatively unpolluted area

**DOI:** 10.1007/s11356-024-35611-w

**Published:** 2024-11-26

**Authors:** Olha Timofieieva, Anna Maria Labecka, Anton Vlaschenko, Alona Shulenko, Ryszard Laskowski

**Affiliations:** 1 Terrestrial Ecosystems and Ecotoxicology Group, Institute of Environmental Sciences, Faculty of Biology, Jagiellonian University, Gronostajowa 7, 30-387 Kraków, Poland; 2 Life History Evolution Group, Institute of Environmental Sciences, Faculty of Biology, Jagiellonian University, Gronostajowa 7, 30-387 Kraków, Poland; 3 Ukrainian Bat Rehabilitation Center, NGO “Ukrainian Independent Ecology Institute”, Kharkiv, 61001 Ukraine; 4 Educational and Research Bat Biology Laboratory, H.S. Skovoroda Kharkiv National Pedagogical University, Valentynivska St., 2, Kharkiv, 61168 Ukraine

**Keywords:** Bioaccumulation, Cadmium, Copper, Zinc, Lead, Ukraine

## Abstract

Bats, as exceptionally long-lived small mammals, are at particular risk of metal poisoning due to the tendency of metals to bioaccumulate throughout their lives. In our study, we investigated the general question of how trace metal concentrations change with age in different bat tissues on the example of *Pipistrellus kuhlii lepidus*, which lives for years in one area and is strongly associated with urban environments. To determine the exact age of the individuals, osteochronology was applied, counting the number of dentine rings in cross-sections of the upper canine tooth of each individual. The age of 57 individuals of *P. kuhlii lepidus*, representing ca. 10% of the colony, was identified. Whole internal organs (liver, kidneys, lungs, and forearm bones) and samples of external tissues (fur and wing membrane) were analyzed for concentrations of Cd, Cu, Pb, and Zn using atomic absorption spectrometry. We found that concentrations of Cd, Pb, and Zn, but not Cu, increase with the age of the bats, but in relatively unpolluted areas, metal concentrations do not reach the level which can cause chronic adverse effects. Nevertheless, due to the confirmed accumulation of metals in bat tissues with age, toxic effects can be expected in older individuals in areas where trace metal concentrations are elevated.

## Introduction

In urbanized and many rural environments, trace metal concentrations can be significantly elevated because of road traffic, mining activities, foundries, smelters, the use of sewage sludge as fertilizers, and other human activities (Wuana and Okieimen 2011). In rural areas, the primary source of trace metals is agricultural activities. Pesticides and fertilizers applied to soil can be an important source of As, Pb, and Cd (Bloemen et al. 1995). Hence, organisms of all trophic levels may suffer from chronic intoxication (Flache et al. 2016), with top predators being potentially particularly jeopardized due to the possible biomagnification of certain metals. Although the bioaccumulation of trace metals in terrestrial food webs has already been investigated, most of the research has focused on domestic herbivores due to the possible health risks to humans (Gall et al. 2015; Robards and Worsfold 1991). The bioaccumulation of trace metals in wildlife is less described, and the majority of studies on the small mammals are done on raccoons, wood mice, field mice, yellow-necked mice, voles, and shrews (Gall et al. 2015). Previous research showed that small terrestrial mammals bioaccumulate trace metals in their tissues when they inhabit abandoned mines, and carnivorous species showed greater accumulation in comparison to omnivores and herbivores (Andrews et al. 1984; Hunter et al. 1989; Ma and Talmage 2001; Shore 1995).

Poisoning even with relatively low doses of some trace metals, such as lead (Pb), mercury (Hg), cadmium (Cd), and arsenic (As), can lead to weakness, uncoordinated movement, and muscle tremors or spasms, as well as sublethal effects at the biochemical, physiological, and histological levels (Burger and Gochfeld 2000; Nawrot et al. 2010; Wren 1986). The excessive accumulation of essential and non-essential metals in mammals can cause oxidative stress, DNA damage, tissue damage, weakening of the immune system (Boyd 2010; Lilley et al. 2013; Ruiz et al. 2019; Zocche et al. 2010), and in some cases cause direct mortality (Clark and Shore 2001). Lead and cadmium belong to the most hazardous metals and can be toxic to the organisms even at low concentrations; copper and zinc are essential metals, which are needed for the normal organism functioning but can be toxic at elevated concentrations (Kabata-Pendias and Mukherjee 2007).

The majority of bat species are insectivorous and occupy the highest food web level, which, combined with their longevity, makes them particularly exposed to the bioaccumulation of pollutants (Zukal et al. 2015). The record lifespan among bats belongs to Siberian whiskered myotis (*Myotis sibiricus*), with a reported maximum lifespan of 41 years (Podlutsky et al. 2005; Zhigalin 2020). As long-lived mammals, bats have a potential as a model species not only for research of aging (Brunet-Rossinni and Austad 2004) but also for monitoring environmental contaminants over time, including trace metals (Zukal et al. 2015).

**Fig. 1:**
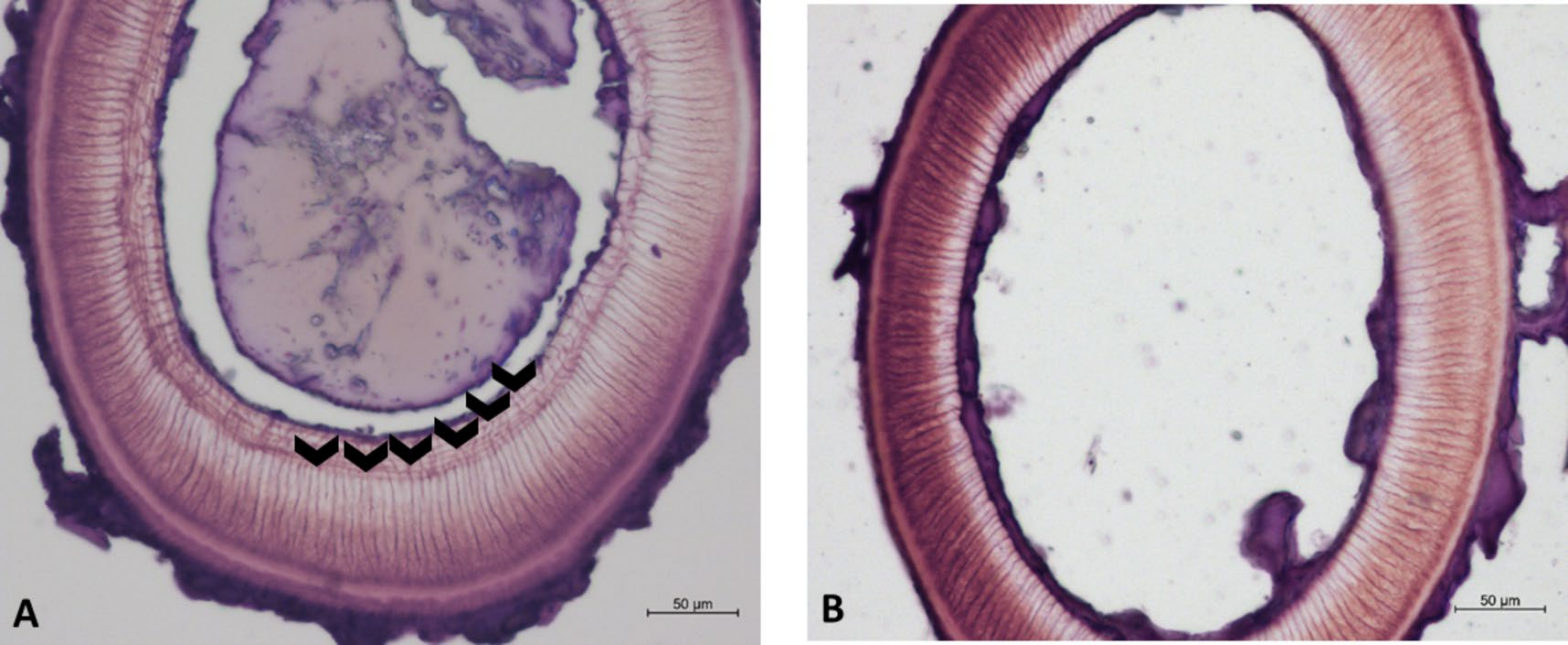
Examples of slides with cross sections of *P. k. lepidus* teeth with different numbers of dentine rings indicated by arrows. **A** Cross section of an individual which is 6 years old (6 dentine rings detected). **B** Cross section of this year born individual (0 dentine rings)

Long-term exposure even to relatively low levels of some metals may result in the gradual development of symptoms. The main obstacle in studies on the accumulation of pollutants with the age of bats is the difficulty in age determination in living animals as it is only possible to tell apart this-year-born individuals from older ones (Brunet-Rossinni and Wilkinson 2009). During the last decades, molecular methods of age estimation have been developed for a few species, but they remain controversial and species-specific (Dunshea et al. 2011; Garde et al. 2010; Wilkinson et al. 2021). Methodologies of age determination based on tooth or bone morphology are still widely used for most animals, especially amphibians and reptiles (Klevezal 1988; Rozenblut and Ogielska 2005). Christian (1956) first used the technique of age identification by counting growth lines in the dentine and cementum of bat teeth for big brown bats (*Eptesicus fuscus*); later, Klevezal and Kleinenberg (1969) described it for greater mouse-eared bats (*Myotis myotis*) and common noctule (*Nyctalus noctula*). Histological analysis showed that the dentine of canine and molar teeth roots forms dark layers each winter (Funakoshi and Uchida 1982), which can be used as an age indicator (Fig. 1).

In this study, we evaluated the bioaccumulation of the four most commonly studied trace metals, cadmium (Cd), copper (Cu), lead (Pb), and zinc (Zn), in the liver, kidneys, forearm bones, muscles, fur, and wing membrane of the eastern taxon of Kuhl’s pipistrelle, *Pipistrellus kuhlii lepidus* (Blyth 1845), bats which were collected from a relatively uncontaminated rural area in eastern Ukraine (Timofieieva et al. 2023). We examined the bats to tell if they tend to accumulate trace metals with age even at such moderate environmental concentrations and, if so, whether this accumulation differs among organs. Given the longevity of bats, the accumulation of trace metals with age would mean that metal pollution could be dangerous and potentially lead to chronic effects in older individuals and ultimately to population decline, perhaps even at moderate exposure levels.

## Materials and methods

### The species and sample collection

Our focal species, *P. kuhlii lepidus,* is a small bat, weighing 5–10 g, with a forearm length of 31–36 mm (Andriollo et al. 2015; Hukov et al. 2020; Sachanowicz et al. 2017). It is an ecologically and behaviourally flexible, adaptable species and can benefit from urbanization (Ancillotto et al. 2015). Its foraging area can be up to 4.5 km, while its home range is less than 2 km^2^ (Amichai and Korine 2020). *Pipistrellus kuhlii* has a maximum recorded lifespan of 8 years (Rakhmatulina 2005), while in other *Pipistrellus* species, maximum recorded lifespan varies between 8 and 16 years (Wilkinson and South 2002). As a resident species*, P. k. lepidus* lives for years in one area and is strongly associated with built-up environments (Hukov et al. 2020), making it of particular interest in terms of potential hazards from pollution. The species is described as having an opportunistic foraging strategy based on the composition of its diet, which is composed primarily of Culicidae and Lepidoptera species but also includes Hymenoptera, Brachycera, Tipulidae, Chironomidae/Ceratopogonidae, and Coleoptera (Cohen et al. 2020; Goiti et al. 2003).

Carcasses of *P. k. lepidus* (*n* = 57) were obtained from the Bat Carcasses Storing Collection of the Bat Rehabilitation Center (BRC) of Feldman Ecopark in Kharkiv (Ukraine). They originated from the group of bats that was rescued from the school building in Karlovka village (49°27′27.4536″ N 35°7′12.0252″ E) in December 2018 during renovation works (Hukov et al. 2020). As shown in our earlier study (Timofieieva et al. 2023), the area can be considered uncontaminated with metals, with the following average metal concentrations in soil dry weight (dw): Cd 0.27 mg/kg, Cu 26.67 mg/kg, Pb 42.75 mg/kg, and Zn 156 mg/kg. In no case, the allowable limits for metal concentrations in agricultural soils, according to Ukrainian law, were exceeded (Timofieieva et al. 2023). Some bats brought to BRC were already dead, and some soon died of exhaustion suffered before reaching the Center. The Center works under the permission of the Kharkiv Oblast Authority of Ecology and Natural Resources, and the Ethical Commission of V.N. Karazin Kharkiv National University. The carcasses were frozen at − 20 °C for subsequent analyses (which included approval of this study).

The liver, kidneys, lungs, forearm bones, and muscles were dissected, and samples of fur and wing membrane were taken. Additionally, we took upper canine teeth from each individual for age determination. All samples, except for the teeth, were oven-dried to the constant weight at 60 °C to enable transportation from Kharkiv to the Institute of Environmental Sciences (IES), Jagiellonian University in Kraków (Poland), for trace metal analysis (Timofieieva et al. 2023).

### Analysis of trace metal concentrations

After being dried at 105 °C for 24 h, the samples from the internal (liver, kidney, forearm bones) and external (wing membrane, fur) organs were weighed to the nearest 0.001 g (Radwag AS 160/C/2, Poland) and then digested in boiling nitric acid (HNO_3_; 69.0–70.0%, Baker Instra-Analyzed, USA), at the ratio of 1:20 (2 ml of the acid per 0.1 g of tissue). After the samples were completely digested and acids evaporated, the samples were dissolved up to 10 ml with deionized water acidified with HNO_3_. Atomic absorption spectrophotometers were used to measure the concentrations of Cd, Cu, Pb, and Zn in each sample. Depending on the concentration, flame (AAnalyst 200, PerkinElmer, USA) or graphite furnace (PinAAcle 900Z, PerkinElmer, USA) atomization was used. The accuracy and precision of the extraction and analytical methods were evaluated by analysing a certified reference material (bovine liver, National Institute of Standards and Technology, USA, Standard Reference Material 1577c) and blank samples (nitric acid only), which were prepared and subjected to the same procedure as the samples. The limits of detection (LoD) were as follows: for Cd 0.024 µg/l, for Cu 0.027 µg/l, for Pb 0.530 µg/l, and for Zn 0.011 µg/l using graphite furnace atomization, and 0.012 mg/l for Cu when using flame atomization. No samples were below the limit of detection for Cu while 12% of samples for Cd and Pb and 14% for Zn were below. Concentrations below the LoD were not considered in the analysis. All concentrations are expressed in mg/kg dw (dry weight).

### Determining the age of bats

Fifty-seven carcasses were taken for analysis. The age of adults was estimated with the skeletochronological procedure described by Goldin et al. (2019), Klevezal (1988), and Rozenblut and Ogielska (2005), using the upper canine tooth of each individual. First, each tooth was fixed with 4% buffered neutral formalin (Chempur, Poland) for 24 h, decalcified in a mix of 4% buffered formalin and 10% formic acid (Chempur) for 3 h, washed with deionized water (15 min × 4 times) and stored in 80% ethanol (P.P.H. ‘Stanlab’ Sp. z. o. o., Poland). Then, each tooth was placed in a cryoembedding medium (Milestone, Italy), frozen on a Peltier plate, and cut into 12-µm-thick slices at − 23 °C using cryostat (CM1950, Leica, Germany). Teeth cross sections were dried at room temperature and then stained with 0.05% water solution cresyl violet acetate (Carl Roth, Germany) for 10–15 min. Slides were analyzed under a light microscope (Eclipse 80i, Nikon, Japan) in a brightfield and photographed using a digital camera (Axiocam 305 color, Zeiss) and image acquisition software (ZEN 3.3. blue edition). For age identification, the dentine rings on the cross sections were counted (Fig. 1). Growth layers in dentine were complex in structure, with the main element usually visible as an intensively stained line of varying width, marking a complete year of life (Goldin et al. 2019; Klevezal 1988).

### Statistics

To test whether and how metal concentrations in bat tissues depend on age, regression analysis was applied using the comparison of regression lines procedure to account for differences between tissues in intercepts and/or slopes. If no significant difference between slopes or intercepts was detected, this term was removed from the model. The results were checked for unusual residuals, the data points with studentized residuals > 3 were removed from the model, and the final model was fitted. In addition, a general linear model (GLM) procedure, with tissue as a categorical factor and age as a quantitative factor, was used to determine the extent to which individual tissues differed in their accumulation of each metal. Means were separated using Tukey HSD intervals.

Metal concentrations were log-transformed for all analyses as data distributions were right-skewed. In all statistical analyses, *p* ≤ 0.05 was adopted as the significance level. The analyses were performed in Statgraphics Centurion 19 (Statgraphics Technologies, Inc.).

## Results

**Fig. 2:**
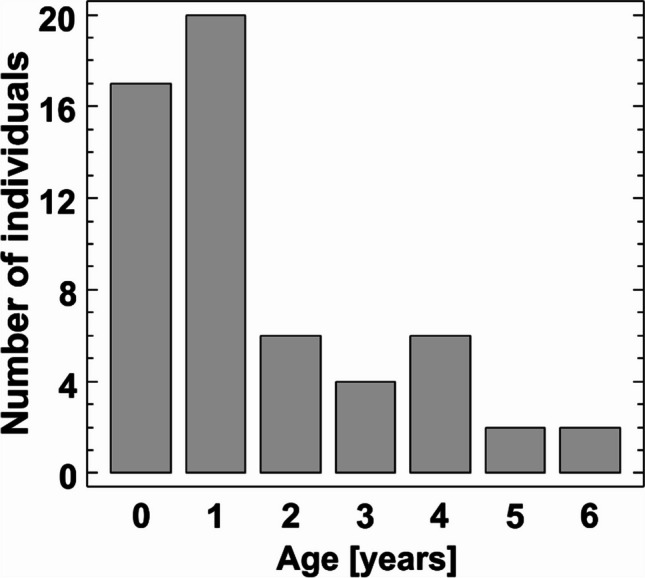
Age structure of *P. k. lepidus* used in the analysis of trace metal concentration in tissues (*n* = 57)

The age of 57 individuals of *P. k. lepidus* was identified. The skeletochronological analysis demonstrated that the majority of investigated individuals were this-year-born and 1 year old. The oldest bats were 6 years old, and the two oldest classes were represented by only two individuals each (Fig. 2). All the bats came from the same wintering group, with a total of 641 individuals (Hukov et al. 2020), meaning that we identified an exact age of approximately 10% of the whole group.

The results of chemical analyses of the certified material showed good agreement between measured and nominal concentrations, with the following recovery values: Cd 96.7%, Cu 95%, Pb 91%, and Zn 90%. Concentrations measured in bat tissues were not corrected for recovery.

**Fig. 3:**
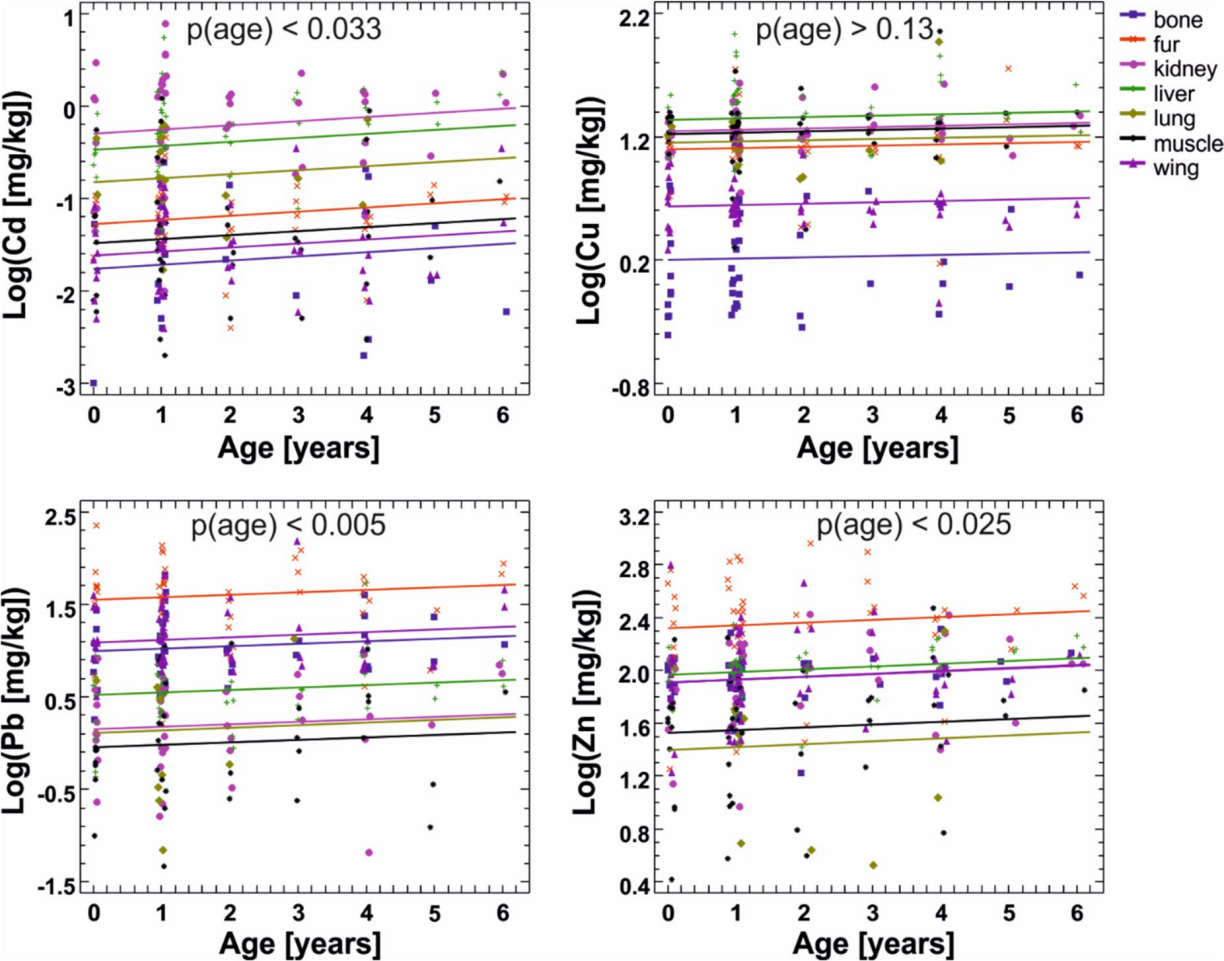
Relationships between age and metal concentrations in tissues of *P. k. lepidus* bats from a rural area (regression analysis applied). No significant differences in slopes were found for any of the metals while the intercepts were different at *p* < 0.0001 in all cases; *p* values for slope (relationship with age) are given on the plots. The data points have been horizontally jittered to minimize overlap—the horizontal spread of concentrations within age class does not represent any real variance with age

The regression models for all metals, with tissue included as a categorical factor, were significant at *p* < 0.0001 but this did not in all cases indicate a significant relationship to age, as a large part of the variance was explained by differences between tissues. Statistically significant differences between tissues were found for concentrations of all metals (intercepts differed at *p* < 0.0001), while the slopes were the same for all tissues (*p* > 0.6). There were three data points with studentized residuals > 3 among concentrations of Cd (out of 323 in total), four in Cu (out of 340), five in Pb (out of 320), and four in Zn (out of 324). The final regression models showed a statistically significant increase in metal concentration with age for Cd, Pb, and Zn but not for Cu (Table 1, Fig. 3). However, in all three cases of a significant increase in metal concentration with age, the rate of increase was very low, and the variance in concentrations of metals within age classes, visible particularly well in the two youngest and most numerous classes, was larger than the variance with age (Fig. 3).

**Table 1:** Regression analysis results for the relationship between age and log-transformed metal concentrations in different tissues. No significant differences in slopes were found between tissues for any of the metals. NS - non-significant

*Metal*	*Model F and p values*	*Model R*^*2*^* (%)*	*Final model ANOVA for variables in the order fitted*		
			*F* and *p* values for age	*F* and *p* values for intercepts	Slope
Cd	*F*_7,312_ = 47.3 < 0.0001	51.5	*F*_1,7_ = 4.62 < 0.033	*F*_6,7_ = 54.4 < 0.0001	0.043
Cu	*F*_7,328_ = 91.9 < 0.0001	66.2	*F*_1,7_ = 2.20 > 0.13	*F*_6,7_ = 106.8 < 0.0001	0.010 (NS)
Pb	*F*_7,307_ = 62.6 < 0.0001	58.8	*F*_1,7_ = 8.10 < 0.005	*F*_6,7_ = 71.7 < 0.0001	0.027
Zn	*F*_7,312_ = 25.7 < 0.0001	36.5	*F*_1,7_ = 5.14 < 0.025	*F*_6,7_ = 29.1 < 0.0001	0.021

**Fig. 4:**
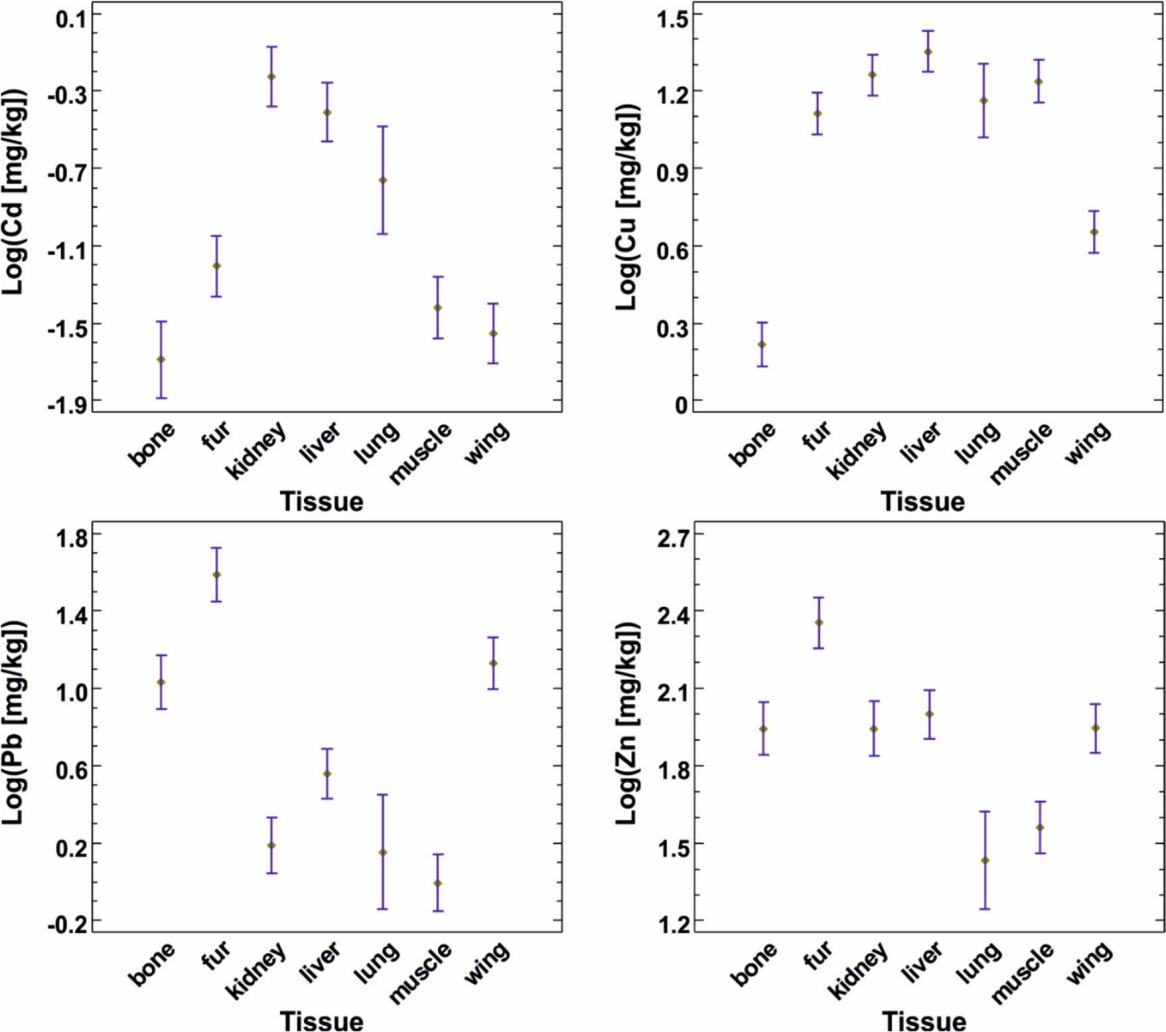
The pattern of metal accumulation in different tissues of *Pipistrellus kuhlii* bats from the rural area of Karlovka (Ukraine). The graphs show the least square means with 95% Tukey HSD intervals for log-transformed concentrations

The four metals accumulated in different tissues in different ways (Fig. 4). Cadmium accumulated mostly in the kidneys, liver, and lungs, copper reached its highest concentrations in the liver, kidney, fur, lungs, and muscles, intermediate in wing membrane, and the lowest in bones. In contrast, lead accumulated mostly in fur, bones, and wing membrane, with lower concentrations in the remaining tissues. The highest concentration of zinc was found in fur, followed by liver, bone, and wing membrane, with lungs and muscles having the lowest concentrations (Table 2).

**Table 2:** Mean metal concentrations with minimum and maximum values in tissues of *P. k. lepidus* from Karlovka village, North-Eastern Ukraine

Tissue	Cd	Cu	Pb	Zn
	[mg/kg]			
Bone	0.057 (*n* = 34) 0.001–0.248	13.31 (*n* = 50) 0.17–549.39	14.41 (*n* = 49) 1.775–66.180	92.6 (*n* = 50) 0.7–203.4
Fur	0.090 (*n* = 53) 0.004–0.451	15.38 (*n* = 53) 1.47–56.60	52.81 (*n* = 51) 1.62–223.58	295.0 (*n* = 52) 9.9–904.7
Kidney	1.145 (*n* = 55) 0.010–7.746	19.65 (*n* = 55) 5.58–43.54	3.56 (*n* = 49) 0.014–58.78	109.8 (*n* = 46) 5.8–424.8
Liver	0.679 (*n* = 57) 0.002–5.420	27.75 (*n* = 57) 0.77–108.16	5.92 (*n* = 57) 0.228–53.58	107.2 (*n* = 56) 1.6–222.9
Lung	0.261 (*n* = 17) 0.017–0.711	18.47 (*n* = 17) 7.25–93.81	7.48 (*n* = 13) 0.02–47.59	48.4 (*n* = 14) 3.4–197.8
Muscle	0.116 (*n* = 52) 0.001–1.197	20.20 (*n* = 53) 0.51–113.30	2.66 (*n* = 46) 0.01–14.64	55.4 (*n* = 51) 2.6–292.6
Wing Membrane	0.049 (*n* = 55) 0.004–0.335	5.41 (*n* = 55) 0.58–17.74	19.22 (*n* = 55) 2.03–151.95	118.3 (*n* = 55) 16.9–632.6

## Discussion

Although the tooth-based age determination method has never been used in *P. k. lepidus*, good results based on counting growth layers have been reported for another *Pipistrellus* species*—P. abramus* (Funakoshi and Uchida 1982). We can assume, thus, that the age of bats determined in this study is trustworthy. The results of the chemical analyses are also reliable, as evidenced by the good agreement between the measured and nominal concentrations in the certified material.

The results presented herein reflect the natural tendency of metals to accumulate in the tissues of bats even in a rather unpolluted area. Our previous study (Timofieieva et al. 2023) showed that mean concentrations of the studied metals in the soil of the agricultural area from which the bats for current research originated were lower than maximum permissible concentrations according to Ukrainian law. Only in one sample did the highest concentration of Zn, 409.5 mg/kg, exceed the maximum allowable level. Although we found a significant increase in concentrations of Cd, Pb, and Zn, the rise was so small that it cannot lead to harmful concentrations in bats inhabiting such relatively unpolluted areas.

We found that lead accumulated mostly in fur, bones, and wing membrane, with lower concentrations in the remaining tissues. According to Ma (2011), kidney lead levels in shrews varied between 3 and 11 mg/kg dw in natural areas on clay or peat and between 13 and 19 mg/kg dw in natural areas on sandy soil, while mice and voles in the respective areas had a kidney lead level of 0.2–0.6 mg/kg dw and 0.4–1.5 mg/kg dw. Such levels of lead in the kidney are not expected to correlate with any toxic effects in mammals. Background concentrations of lead in the bones of mice and voles living in unpolluted sites were in the range of 2–3 mg/kg dw, and the lowest level of bone lead that has been reported for wild populations of shrews is 12 mg/kg dw (Ma and Talmage 2001). Lead in bone is a reliable biomarker of long-term lead contamination since it represents cumulative dose. Shrews’ femur bones in lead-polluted areas had an average lead concentration of 550 mg/kg dw, with some kidney-damaged individuals having a maximum of ca. 1500 mg/kg dw (Ma 2011). With the highest lead concentration detected in our study in bat bones at 66.18 mg/kg dw and the mean of 14.41 mg/kg, the studied bats do not appear to be at risk of lead poisoning. However, the highest concentrations of lead in our study were found in the fur, with an average of 52.81 mg/kg dw and a maximum of 223.58 mg/kg, which is higher than the highest Pb concentration in bat fur found in the literature (148 mg/kg dw., the mean of a pool of 30 specimens of European free-tailed bat; Andreani et al. 2019; Giunta et al 2024). The mean concentration of lead in the livers of bats was 5.92 mg/kg dw and in kidneys 3.57 mg/kg dw; according to Ma (1989) and Shore and Douben (1994), these values do not exceed the lead concentrations that are critical for small mammal kidneys (25 mg/g, dw) or liver (60 mg/kg, dw). For comparison, a mean concentration of 21 mg/kg dw of Pb was found in the livers of four big brown bats in a study on bats affected by white-nose syndrome in New York and Connecticut, USA (Courtin et al. 2010). However, the authors noted that no microscopic lesions indicative of toxicity were observed in the epidermis. Finally, the effects of lead poisoning, such as renal inclusion bodies, were investigated in a black flying fox in Queensland (Skerratt et al. 1998). Reported concentrations were 370 mg/g in the kidney and 16.76 mg/kg in the liver for an adult, and 123.85 mg/kg in the liver and 19.26 mg/kg in the kidney for its pup.

Cadmium accumulated mostly in the kidneys and liver of the studied bats. According to Ma et al. (1991), the critical cadmium concentration in the liver and kidneys of voles *Microtus agrestis* (Rodentia) is 20–30 mg/kg dw and 119 mg/kg dw, respectively. In our research, maximum cadmium concentrations were 7.75 mg/kg dw in the kidney and 5.42 mg/kg dw in the liver, which are both well below the concentrations considered critical in rodents. Unfortunately, no relevant data exist for bats or other insectivorous small mammals (Giunta et al 2024). A single laboratory study examined the effects of directly injecting trace elements into bats (Dixit and Lohiya 1974). Male rat-tailed bats (*Rhinopoma kinneari*) given subcutaneous cadmium chloride injections showed testicular necrosis and seminiferous tubule shrinkage, though the effects were more severe in treated rats. Unfortunately, concentrations in the bat tissues were not reported in the study, so we cannot compare the concentrations with the results from our research. In addition, we did not examine testicular tissue.

Copper and zinc are essential trace metals which are needed for normal functioning of organisms. However, these essential elements are toxic at high concentrations. Copper reached the highest concentrations in the liver and kidneys, while the highest concentration of zinc was found in the fur, followed by the liver. Copper is a component of several proteins and metalloenzymes, and the liver is a storage site of copper (Kabata-Pendias and Mukherjee 2007). The highest concentration of copper in the liver of bats was 108.16 mg/kg dw and in kidneys 43.54 mg/kg dw, which is not considered toxic compared with the concentrations for shrews from contaminated areas (Ma 1987). According to Kabata-Pendias and Mukherjee (2007), under normal conditions, the average copper concentrations in mammalian tissues range from 1.7 to 196 mg/kg dw, with the highest level in the liver and the lowest in the skin.

Zinc concentrations in mammalian tissues typically range from 13 to 210 mg/kg dw, with the skin having the lowest concentrations and the kidneys and liver the highest (Kabata-Pendias and Mukherjee 2007). In our samples, the mean concentration of zinc in the liver was 107.2 mg/kg dw. This value is lower than 182 mg/kg dw found by Cooke et al. (1990) in livers of the insectivorous *Sorex araneus* from contaminated areas but can be considered elevated. Also, Zn concentrations in fur (mean 295 mg/kg dw) appear to be elevated, as according to Hickey et al. (2001), bats from an unpolluted area in Canada had a mean value of 107.6 mg/kg Zn, while bats from a mining area had 314.6 mg/kg.

Although, in general, we did not find concentrations of metals in bat tissues high enough to pose a health risk to bats; the confirmed significant increase in the concentration of metals in bat tissues with age may be an issue in metal-contaminated areas. Indeed, the studies conducted in Southern Brazil showed that bats (*Eptesicus diminutus, Molossus molossus, Tadarida brasiliensis*) from a coal mining area accumulated more metals than those from unpolluted area, and one species showed DNA damage in the mining area (Zocche et al. 2010). Concentrations of Cd, Pb, and Zn in bat livers in our research are lower than in bats from Southern Brazil, which faced DNA damage. Additionally, compared to bats from non-contaminated areas in the Northeastern USA, individuals captured in mercury-contaminated areas had much higher concentrations of mercury in their blood and fur (Yates et al. 2014). Hill et al. (2018) confirmed that bats (*Neoromicia nana*) can bioaccumulate trace metals, such as arsenic, in their brain tissue in the metal-contaminated areas, which also means that arsenic can cross the protective brain barrier.

Higher concentrations of trace metals in adult bats have been reported previously. For instance, adults of *Myotis myotis* had higher concentrations of Pb in their muscles than juveniles (Pikula et al. 2010) and significantly higher Hg concentrations were found in the fur of adult Chinese noctules (*Nyctalus plancyi*) and in the blood of *Myotis* sp. (Heiker et al. 2018; Yates et al. 2014). However, if our study reflects well the age structure of populations of insectivorous bats, it seems that in unpolluted environments only very few oldest individuals can be endangered by harmful metal concentrations due to their accumulation with age. For young individuals, which make up the vast majority of bat populations, the potential biomagnification of metals along trophic chains may be a more important concern. It would thus be valuable to extend such studies to areas where trace metal concentrations are clearly elevated, such as large cities with high traffic and surroundings of mines and smelters, and to assess the transfer of trace metals in the bat trophic chain.

Several previous studies on mammals have shown that if wild animals are exposed to metal pollution, they tend to bioaccumulate metals as they age. Studies on the European roe deer (*Capreolus capreolus*), for instance, have demonstrated a strong age dependency of trace metal concentrations (Frøslie et al. 1984; García et al. 2011). This species has been identified as a good bioindicator species of trace metal pollution (Fan 1996). Medium-sized carnivorous, including red foxes (*Vulpes vulpes*), which are at the top of the food chain and vulnerable to biomagnification of pollutants (Bilandzic et al. 2010), also were used as bioindicator species. In foxes, an increase of trace metals in the tissues also was observed with age (Cybulski et al. 2009; Naccari et al. 2013). It seems, thus, that the phenomenon observed in this study in the bats, i.e., the increasing concentrations of Cd, Cu, and Zn with age, is nothing exceptional. What makes this group of mammals unique, however, is their particular longevity combined with the accumulation of metals with age, which can lead to toxic levels in older individuals in contaminated environments.

## Conclusions

The concentrations of cadmium, copper, lead, and zinc found in bat tissues were below the values considered toxic for mammals. However, we found a statistically significant increase in concentrations of Cd, Pb, and Zn, but not Cu, with age, which may mean that in the areas where trace metal concentrations are elevated, such as large cities with heavy traffic and surroundings of mines and smelters, older bats may bioaccumulate metals and be at risk of chronic toxic effects. Due to the confirmed accumulation of metals in bat tissues with age, further research focusing on the transfer of trace metals in food chains, especially in polluted environments, is needed. It would be also important to include histopathological and haematological analyses in future research.

## Data Availability

Data on trace metal (Cd, Cu, Pb, and Zn) concentrations in the tissues of age-identified *Pipistrellus kuhlii lepidus* from an unpolluted area are available on Zenodo (Timofieieva et al. 2024) with the identifier 10.5281/zenodo.14007172.
